# Advancing osteochondral tissue engineering: bone morphogenetic protein, transforming growth factor, and fibroblast growth factor signaling drive ordered differentiation of periosteal cells resulting in stable cartilage and bone formation in vivo

**DOI:** 10.1186/s13287-018-0787-3

**Published:** 2018-02-21

**Authors:** L. F. Mendes, H. Katagiri, W. L. Tam, Y. C. Chai, L. Geris, S. J. Roberts, F. P. Luyten

**Affiliations:** 1 Tissue Engineering Laboratory, Skeletal Biology and Engineering Research Center, KU Leuven, Campus Gasthuisberg O&N 1, Herestraat 49, bus 813, 3000 Leuven, Belgium; 2 Prometheus, Division of Skeletal Tissue Engineering, KU Leuven, O&N 1, Herestraat 49, bus 813, 3000 Leuven, Belgium; 3 Biomechanics Research Unit, University of Liege, Chemin des Chevreuils 1 – BAT 52/3, 4000 Liege 1, Belgium; 4 Biomechanics Section, KU Leuven, Celestijnenlaan 300C bus 2419, 3001 Leuven, Belgium; 5 Institute of Orthopaedics and Musculoskeletal Science, Division of Surgery & Interventional Science, University College London, The Royal National Orthopaedic Hospital, Stanmore, Middlesex, HA7 4LP UK

**Keywords:** Periosteal cells, Osteochondral defect, Cartilage tissue engineering, Growth factors, Subchondral bone regeneration

## Abstract

**Background:**

Chondrogenic mesenchymal stem cells (MSCs) have not yet been used to address the clinical demands of large osteochondral joint surface defects. In this study, self-assembling tissue intermediates (TIs) derived from human periosteum-derived stem/progenitor cells (hPDCs) were generated and validated for stable cartilage formation *in vivo* using two different animal models.

**Methods:**

hPDCs were aggregated and cultured in the presence of a novel growth factor (GF) cocktail comprising of transforming growth factor (TGF)-β1, bone morphogenetic protein (BMP)2, growth differentiation factor (GDF)5, BMP6, and fibroblast growth factor (FGF)2. Quantitative polymerase chain reaction (PCR) and immunohistochemistry were used to study *in vitro* differentiation. Aggregates were then implanted ectopically in nude mice and orthotopically in critical-size osteochondral defects in nude rats and evaluated by microcomputed tomography (µCT) and immunohistochemistry.

**Results:**

Gene expression analysis after 28 days of in vitro culture revealed the expression of early and late chondrogenic markers and a significant upregulation of NOGGIN as compared to human articular chondrocytes (hACs). Histological examination revealed a bilayered structure comprising of chondrocytes at different stages of maturity. Ectopically, TIs generated both bone and mineralized cartilage at 8 weeks after implantation. Osteochondral defects treated with TIs displayed glycosaminoglycan (GAG) production, type-II collagen, and lubricin expression. Immunostaining for human nuclei protein suggested that hPDCs contributed to both subchondral bone and articular cartilage repair.

**Conclusion:**

Our data indicate that in vitro derived osteochondral-like tissues can be generated from hPDCs, which are capable of producing bone and cartilage ectopically and behave orthotopically as osteochondral units.

**Electronic supplementary material:**

The online version of this article (10.1186/s13287-018-0787-3) contains supplementary material, which is available to authorized users.

## Background

Osteochondral and joint surface defects account for up to 60% of total knee arthroscopies performed in the United States [1, 2]. Besides pain and functional impairment, localized joint defects are a clear risk factor for the development of osteoarthritis (OA) when left untreated [3–5]. In the United States, 9% of the total population older than 30 years suffers from OA of the hip or knee, where approximately 12% of cases arise secondary to joint trauma [6]. The poor regenerative capacity of articular cartilage and a rather limited understanding of articular cartilage neogenesis have hampered the development of successful therapies to restore joint surface defects. Bone marrow stimulation techniques, such as microfracture, have been widely applied in cases of chondral and osteochondral injury. However, long-term results indicate that the reparative tissues consist mostly of fibrocartilage, resulting in eventual cartilage degeneration and thus reintervention [7]. Fresh osteochondral grafts have provided positive outcomes when applied to large osteochondral defects, but a shortage of appropriate tissue is a clear limitation towards general clinical application [8]. Also, decellularized osteochondral allografts display high failure rates in the treatment of large osteochondral defects [9]. Therefore, numerous cell-based therapies have been attempted to address these limitations. The clinical use of cell-based therapies for cartilage repair rely on autologous chondrocytes that are harvested from a low load-bearing area of the patient’s own knee and transplanted into the defect area (autologous chondrocyte implantation (ACI)), with or without the use of supportive scaffolds [10]. Indeed, standard ACI is relatively successful in isolated deep cartilage defects in otherwise healthy/young joints, but it is not appropriate for osteochondral defects [11]. Mesenchymal stem cell (MSC)-based therapies have also been attempted; however, clinical trials report a general inefficacy when considering functional recovery of damaged joint surfaces [12–14]. Indeed, to date, only a few studies have claimed in vitro differentiation of MSCs towards the stable, articular cartilage phenotype [15–18].

The bone periosteum has long since been investigated for cartilage and joint surface repair. Originally, the ACI procedure involved the bone periosteum as a protective, biologically active membrane that had positive paracrine effects on implanted chondrocytes [19]. Due to the abundance of skeletal progenitor cells in the periosteum, periosteal graft transplantations were also investigated for osteochondral defect repair. However, despite qualitative positive outcomes, data were inconsistent [20, 21]. Considering the joint’s architecture, particularly the complex interface between cartilage and subchondral bone, it remains a challenge to generate cell-based constructs that not only allow for cartilage and bone formation, but also possess enough plasticity to operate a regenerative process orchestrated by the different microenvironments present in the joint. As recently reviewed [22], more mature cartilage constructs display attractive properties for the treatment of cartilage defects due to their increased resistance to proinflammatory cytokines and their capacity to recruit or commit neighboring cells to the regenerative process. These properties are provided by their cartilaginous extracellular matrix (ECM) and may represent a significant advantage to the microfracture and ACI techniques.

Considering the presence of osteo- and chondroprogenitor cells in the periosteum, it was hypothesized that human periosteum-derived progenitor cells (hPDCs) could generate tissue intermediates (TIs) which, upon implantation in proper environment, would allow the formation of a stable layer of cartilage and a deep layer of new endochondral bone. Herein, we show the self-assembling and osteochondral potential of hPDCs cultured in vitro for 4 weeks using a novel growth factor (GF) cocktail [23]. When implanted ectopically in nude mice, TIs were able to generate multiple tissues, including mineralized cartilage, bone, and bone marrow. When implanted into osteochondral defects in rats we found an intrinsic osteochondral regenerative potential, suggesting the clinical relevance of this protocol for joint surface repair.

## Methods

### Periosteal cell isolation and culture

hPDCs were isolated from periosteal biopsies of different donors undergoing distraction osteogenesis surgeries (*n* = 4; two male and two female donors, aged 28.7 ± 12.3 (mean ± SD) years old) as previously described [24]. Briefly, the periosteum was stripped from the tibia, minced, and digested overnight at 37 °C in type IV collagenase (440 units/mg; Invitrogen, Merelbeke, Belgium) in growth medium (high-glucose Dulbecco’s modified Eagle’s medium (DMEM; Invitrogen, Merelbeke, Belgium) supplemented with 10% fetal bovine serum (FBS; BioWhittaker, Verviers, Belgium), and an antibiotic–antimycotic solution (100 units/ml penicillin, 100 μg/ml streptomycin, and 0.25 μg/ml amphotericin B; Invitrogen, Merelbeke, Belgium). A pool of hPDCs was then created based on identical growth kinetics in vitro and in vivo bone forming capacity. The ethical committee for Human Medical Research (KU Leuven) approved all procedures, and patient informed consent forms were obtained.

### Cell culture and chondrocyte differentiation

Cultures of hPDCs were prepared as previously described [25]. Briefly, 20-μl droplets of a cell suspension containing 20 × 10^6^ cells/ml were seeded in 24-well plates and incubated at 37 °C in 95% humidity for 3 h. These micromasses were allowed to deposit ECM and to develop cell-to-cell, cell-to-matrix, and cell-to-plastic interactions in a three-dimensional environment for the course of 28 days. To induce chondrocyte differentiation, cells were cultured with 500 μl of chemically defined chondrogenic medium [26] consisting of LG-DMEM (Gibco, UK) supplemented with 100 μM ascorbate-2-phosphate, 100 nM dexamethasone, 40 μg/ml proline, and insulin-transferrin-selenium (ITS) + premix universal culture supplement (BD Biosciences, Bedford, MA), including 6.25 mg/ml insulin, 6.25 mg/ml transferrin, 6.25 mg/ml selenious acid, 1.25 mg/ml bovine serum albumin (BSA), and 5.35 mg/ml linoleic acid. This basal chondrogenic medium (BCM) was supplemented with 10 ng/ml transforming growth factor (TGF)-β1, 100 ng/ml bone morphogenetic protein (BMP)2, 100 ng/ml growth differentiation factor (GDF)5, 0.2 ng/ml fibroblast growth factor (FGF)2, and 1 ng/ml BMP6, as described elsewhere [23]. Rho kinase inhibitor Y27632 [27] (20 μM) was used to minimize cytoskeletal tension and prevent cell detachment and the formation of a necrotic center. This medium is referred as “GF cocktail”. Culture medium was refreshed every 2 days. Negative control aggregates (CTRL) were cultured in BCM with no supplementation of GFs. For osteochondral defect implantation, positive control aggregates of hPDCs were cultured in BCM supplemented with 20 μM Rho kinase inhibitor Y27632 and 10 ng/ml of TGF-β1 for 28 days (TGF-β1 aggregates).

Healthy human articular chondrocytes were obtained from patients undergoing hip replacement for osteoporotic or malignancy-associated fractures. Briefly, the cartilage was sliced into pieces of 4 × 4 mm before being washed three times in 1% (vol/vol) antibiotic–antimycotic/phosphate-buffered saline (PBS) and incubated with 1 mg/ml pronase (Roche)/DMEM-F12 at 37 °C for 30 min at slow rotation (100 rpm). After overnight incubation with 1 mg/ml collagenase B (Roche)/DMEM-F12 at 37 °C, chondrocytes were filtered through a 70-μm cell strainer (Corning), washed twice with PBS, seeded at 1 × 10^6^ cells/T75 flask and cultured for 7–14 days in maintenance medium (DMEM-F12, containing 1% (vol/vol) antibiotic–antimycotic (Gibco), 10% FBS (Gibco), and 5% l-glutamine (Thermo scientific)). Passage 2 cells were used for experiments. The University Hospitals Leuven Ethics Committee and Biobank Committee approved the study, and specimens were taken with patients’ informed consent.

### In vivo ectopic and orthotopic bone and cartilage formation assays

To study ectopic bone and cartilage forming capacity, TIs were cultured in chondrogenic medium supplemented with 10 ng/ml TGF-β1, 100 ng/ml BMP2, 100 ng/ml GDF5, 0.2 ng/ml FGF2, and 1 ng/ml BMP6 for 28 days, and transplanted subcutaneously into nude mice following an adapted version of a previously described protocol [28]. Briefly, TIs were gently lifted using a plastic cell scraper before being washed twice in PBS at room temperature and then being transplanted onto the subcutaneous tissue of nude mice (*n* = 8; 11 weeks, male, NMRI^nu/nu^). Mice were sacrificed 2, 4, and 8 weeks after implantation and the explants were immediately incubated in 4% paraformaldehyde (PFA) for 1 h at room temperature before being washed in sterile PBS and preserved at 4 °C.

For orthotopic implantation, TIs were transplanted in critical-size osteochondral defects (1.4 mm diameter × 1 mm depth) of athymic nude rats (*n* = 9; 11 weeks; male; Foxn1nu). One cylindrical osteochondral defect was created 2 mm from the top of the intercondylar notch on the femoral trochlear groove in the knee of each rat. After wound closure, the rats were allowed to walk freely in the cage before being sacrificed at 8 weeks. Three experimental conditions were tested in this model: 1) critical-size osteochondral defects treated with TIs; 2) critical-size osteochondral defects treated with TGF-β1 aggregates (see above); (3) critical-size osteochondral defects that received no treatment and are referred to as “empty defect”.

All animal experimental procedures were approved by the local ethical committee for animal research (KULeuven). The animals were housed according to the guidelines provided by the Animalium Leuven (KULeuven).

### Histochemical and immunohistochemical characterization

Ectopic explants and in vitro cultured TIs were fixed for 1 h in 4% PFA before being paraffin embedded and sectioned (5 μm) for histochemical characterization. The osteochondral defects were fixed overnight in 4% PFA. For alcian blue staining, samples were deparaffinized and rehydrated in methanol before being incubated with 1% alcian blue solution (pH 1) for 1 h and the nuclei counterstained with Nuclear Fast Red for 10 min followed by dehydration in graded ethanol and mounting. For hematoxylin and eosin (H&E) staining, samples were deparaffinized as described above and immersed in Mayer’s hematoxylin solution for 5 min before being washed for 5 min in tap water and incubated for 7 min in eosin solution. Samples were then dehydrated in graded ethanol and mounted.

Immunohistochemistry was performed using rabbit:anti-human antibodies against COL2A1 (1:200; Millipore, Germany), Col1A1 (1:200; ThermoScientific, USA), and Prg4 (1:300; Abcam, UK), guinea pig:anti-human specific against osteocalcin (1:200; kindly provided by Prof. Jeroen Aerssens, KULeuven, Belgium) and mouse:anti-human nuclei (1:100; Millipore, Germany). Histological sections were dewaxed, rehydrated, and subjected to an antigen retrieval step (Additional file 1: Table S1). Endogenous peroxidase activity was quenched with 3% H_2_O_2_ (15 min). Nonspecific binding was blocked using 5% BSA (Sigma, USA) in Tris-buffered saline and 0.1% Tween 20 (TBST; Sigma, USA) for 30 min. Antibodies were diluted in TBST (2.5% BSA) and incubated overnight at 4 °C. Sections were then washed in TBST and incubated for 40 min with TBST (5% BSA). Samples were subsequently incubated with horseradish peroxidase (HRP)-conjugated anti-rabbit, anti-mouse, and anti-guinea pig secondary antibodies (Jackson Laboratory, USA; 1:500) in TBST (2.5% BSA) for 30 min at room temperature. After washing, sections were incubated with DAB+ substrate (Dako, USA) for detection of HRP enzymatic activity. Nuclei were counterstained with Mayer’s hematoxylin followed by sample dehydration in graded ethanol and mounting. Samples were analyzed using the Leica DMR microscope (Leica, Germany) and images were acquired and analyzed with Spot Software V5.1 (SPOT Imaging Solutions, USA).

### Bone and mineralized tissue quantification and analysis

Explanted TIs were scanned at a pixel size of 2 μm using a Phoenix NanoTom S (GE Measurement and Control Solutions) equipped with a diamond target operated at a voltage of 70 kV and a current of 100 mA. A 0.5-mm aluminum filter was used to reduce beam hardening. Reconstruction was performed using the Datos X software and quantification of mineralized tissue using the CTAn software (both from Bruker-μCT, Belgium). CTVox (Bruker-μCT, Belgium) was used for three-dimensional visualization. Explanted knees were scanned at a pixel size of 4 μm using the system and settings described above, with an adjusted current value of 120 mA. After reconstruction, a cylindrical region of interest (ROI) of 1.5 mm in diameter and 1 mm length within the regenerating site was analyzed using the CTAn software (both from Bruker-μCT, Belgium). Three contralateral noninjured knees were also scanned and used as positive controls for bone quantification and characterization within the ROI. The bone volume fraction was estimated by bone volume per tissue volume (BV/TV), the trabecular structures evaluated by trabecular thickness (Tb.Th), the average separation of the trabeculae by trabecular separation (Tb.Sp), and the average number of trabeculae by trabecular number (Tb.N). The statistical analysis was performed using one-way analysis of variance (ANOVA), and statistically significant differences between groups were further investigated using Tukey’s multiple comparison test. Results were considered statistically different for *P* values lower than 0.05.

### Total RNA extraction and quantitative reverse transcription polymerase chain reaction (qRT-PCR) analysis

Total RNA from each construct was isolated using Qiagen RNeasy extraction kit (Qiagen, Germany) and quantified using Nanodrop ND-1000 spectrophotometer (Thermo Scientific). Isolation of total RNA, synthesizing complementary DNA, and running qPCR were performed as described previously [23]. Each sample was tested in duplicate and compared with the housekeeping gene hypoxanthine guanine phosphoribosyltransferase (HPRT1), thus allowing normalization of results. Relative differences in expression were calculated using the 2 − ΔCT method. The statistical analysis was performed using Kruskal-Wallis test. Statistically significant differences between groups were further investigated using Dunn’s multiple comparison test. Results were considered statistically different for *P* values lower than 0.05.

## Results

### hPDCs differentiate into the chondrogenic lineage and generate bilayered tissue intermediates in vitro

**Fig. 1:**
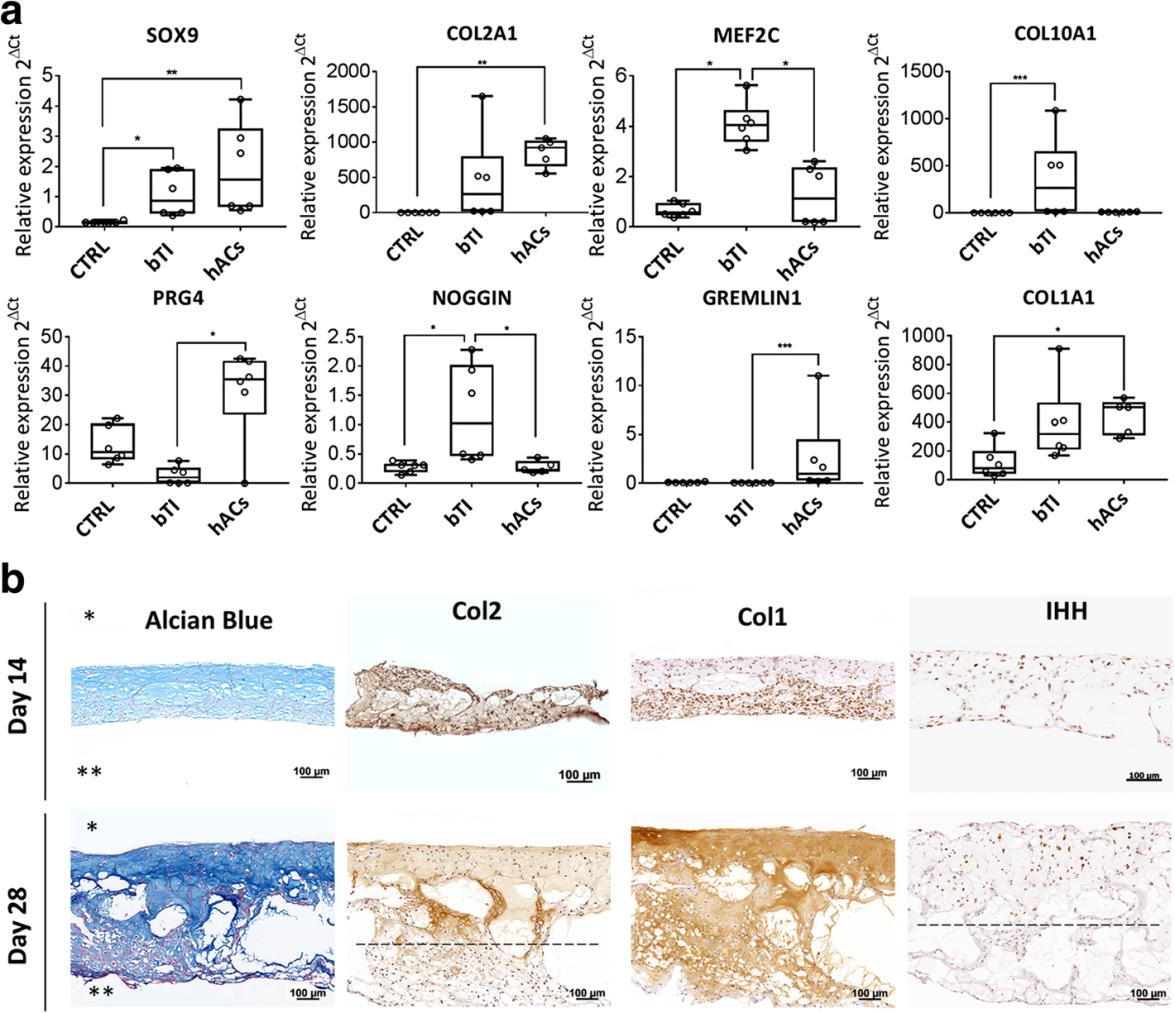
Characterization of TIs at the gene expression and histological levels. **a** mRNA quantification by qPCR of chondrogenic genes in tissue intermediates (TIs), control aggregates (CTRL, no GFs) and human articular chondrocytes (hACs). **b** Longitudinal histological sections (5 μm) of TIs stained for glycosaminoglycans (alcian blue), type-II collagen, type-I collagen, and Indian Hedgehog (IHH) at 14 and 28 days after in vitro culture. Top and bottom layers of TIs are identified by * and **, respectively. Dashed lines represent a critical depth at which cells no longer express type-II collagen or IHH, from top to bottom. Results are representative of two independent experiments. Each experiment was performed using 2–3 technical replicates. In **a**, error bars are max/min; *P < 0.05, **P < 0.01, ***P < 0.001

To define the stage of differentiation after in vitro culture, the relative expression of chondrogenic genes was compared between conditions (Fig. 1a). After 28 days, SOX9 and COL2A1 mRNA levels were higher in TIs compared with control aggregates (CTRL); however, expression of both genes was lower when compared with human articular chondrocytes (hACs). The expression of the hypertrophic genes MEF2C and COL10A1 was significantly upregulated in TIs when compared with CTRL; however, only MEF2C displayed a significant increase in TIs when compared with hACs. The expression of Prg4, whose protein (lubricin) is essential for proper joint lubrication and function, was expressed in hACs and CTRL (with no significant difference) but was significantly downregulated in TIs. The BMP antagonist NOGGIN was equally expressed between hACs and CTRL, but significantly upregulated in TIs. Gremlin1, also a BMP antagonist, was significantly downregulated in both TIs and CTRL compared with hACs. The expression of type 1 collagen was similar between TIs and hACs, suggesting dedifferentiation of the hACs, but significantly lower in CTRL when compared with hACs.

Immunohistochemical characterization of TIs was performed to investigate the spatiotemporal expression of glycosaminoglycans (GAGs), type-II collagen, type-I collagen, and Indian Hedgehog (IHH) (Fig. 1b). A bilayered construct comprising of at least two phenotypically different cell populations was identified after 14 days in culture, evolving to a more evident layered construct by day 28. Alcian blue staining and immunohistochemistry for type-II collagen revealed deposition of both GAGs and collagen at day 14 and day 28; however, type-II collagen deposition and the typical chondrocyte morphology were restricted to the top cell layers of the construct at day 28 (approximately 262 μm ± 55) (top layers in Fig. 1b indicated by *). Type-I collagen deposition was identified at the bottom layers by day 14 (bottom layer in Fig. 1b indicated by **) but had spread throughout the constructs by day 28. IHH-positive cells were heterogeneously distributed at day 14; however, these were restricted to the top layers by day 28, confirming the existence of a preferred location for chondrocyte differentiation. Histological results and gene expression analysis were consistent among two independent experiments.

Overall, these data revealed the potential self-assembling capacity of hPDCs and their different chondrogenic commitment across the construct when cultured in this novel GF cocktail, thus allowing the formation of multiphased tissue intermediates.

### Bone and stable mineralized cartilage are formed after ectopic implantation

**Fig. 2:**
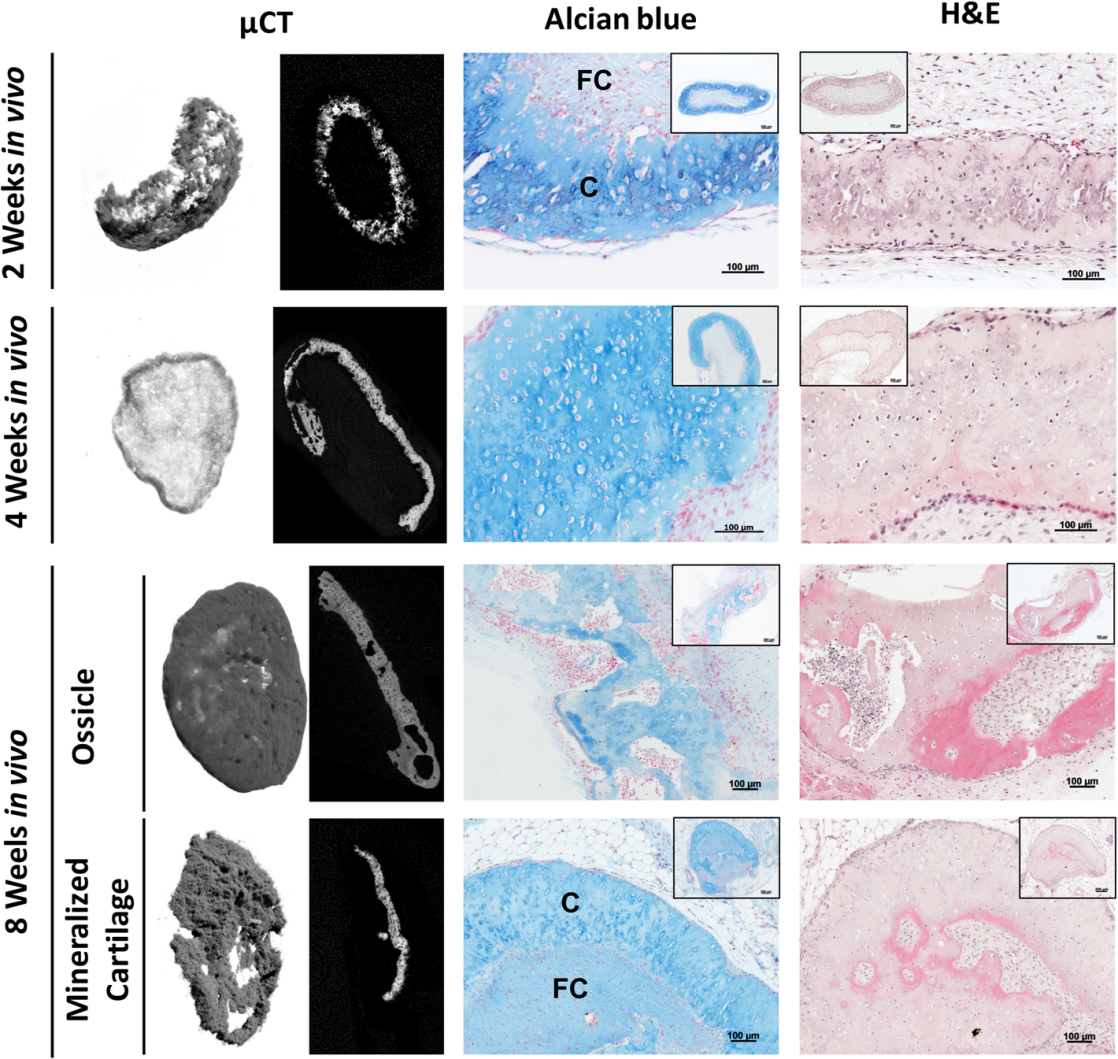
Histological and microcomputed tomography (μCT) analysis of in vitro cultured and ectopically implanted TIs. μCT 3D rendering and cross-sections at the central zone of TIs at 2, 4, and 8 weeks showed extracellular matrix mineralization upon implantation. Histological analysis by alcian blue and hematoxylin and eosin (H&E) staining showed maintenance of fibrocartilage (FC) and chondrocyte-like cells (C) embedded in a GAG-positive matrix at 2 and 4 weeks after implantation. At 8 weeks after implantation, different tissues were formed, namely ossicle-like structures (2/6) and mineralized cartilage showing either low or no bone and bone marrow formation. Results are representative of two independent experiments, each experiment was performed in triplicate

After 28 days of differentiation, TIs were implanted into the dorsal region of nude mice to study their osteoinductive and chondroinductive properties in vivo. Microcomputed tomography (μCT) analysis revealed mineralization of the ECM as early as 2 weeks postimplantation (Fig. 2). Alcian blue and H&E staining performed on the explants at 2 and 4 weeks postimplantation confirmed their chondrogenic phenotype. H&E staining performed at 8 weeks postimplantation revealed that TIs could either form bone ossicles (2/6), displaying bone, cartilage, and bone marrow cavities, or remain as stable mineralized cartilage tissue (4/6) displaying either low or no bone-forming capacity. Alcian blue staining confirmed the presence of only a few remnants of cartilage in the explanted ossicles. Conversely, mineralized cartilage expressed and deposited GAGs in its ECM. Mineralization in vivo appears to occur primarily in the Col2- and IHH-positive areas of the construct, as defined by correlation of the 2-week histology and μCT data (Additional file 2: Figure S1). At later time points, it is possible that IHH/Col2-negative regions may also mineralize, but this is difficult to assess due to the continuous contribution of the host cells to new tissue formation.

**Fig. 3:**
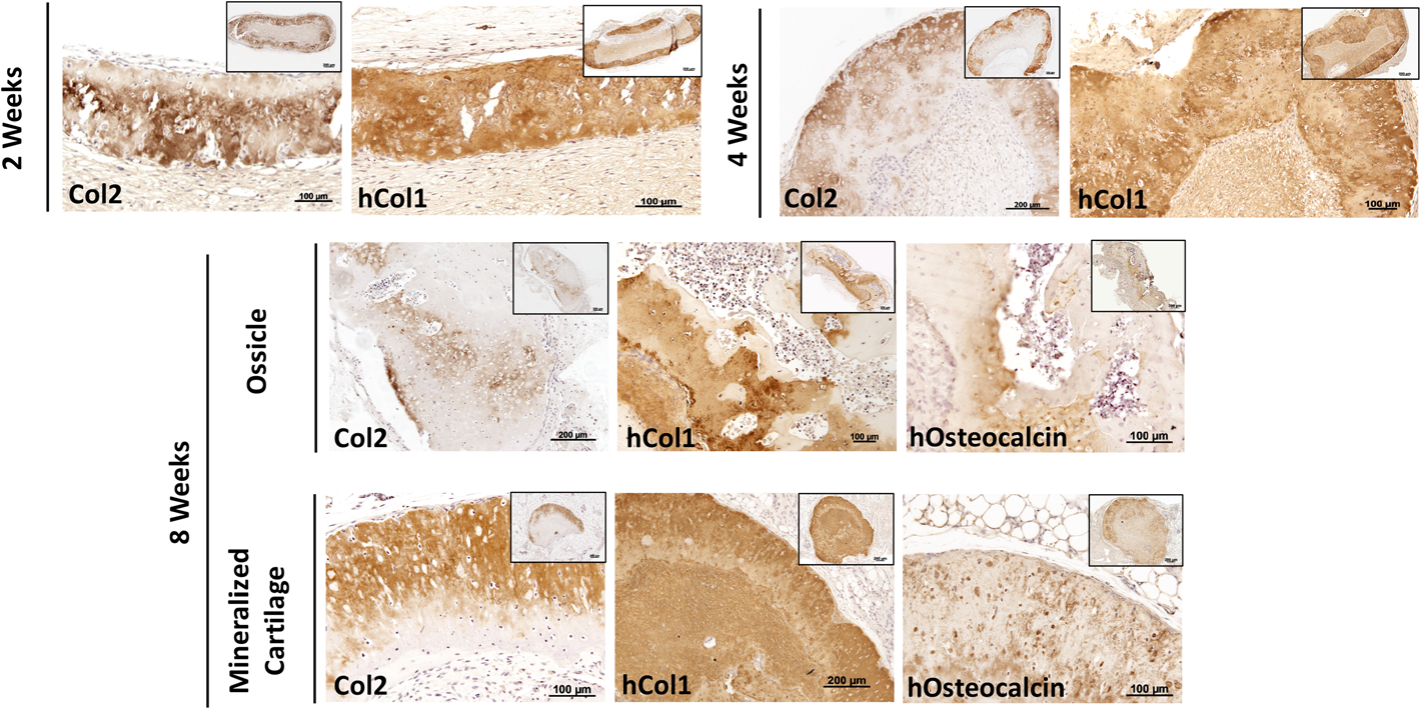
Immunohistological analysis of type-I and type-II collagen deposition and contribution of human (h) cells to bone and cartilage formation by TIs. Immunohistochemistry showed co-deposition of type-II (Col2) and type-I (Col1) collagens at 2 and 4 weeks after implantation. At 8 weeks after implantation, ossicle-like structures showed reminiscences of type-II collagen deposition and contribution of human osteocalcin-positive cells to new bone tissue formation. Mineralized cartilage maintained a dual expression of type-I and type-II collagens and showed positive osteocalcin immunostaining. Results are representative of two independent experiments, each experiment was performed in triplicate

The chondrogenic and osteogenic nature of the explants was further confirmed by immunohistochemistry. Type-I collagen was deposited throughout the constructs at 2 and 4 weeks postimplantation. In contrast, type-II collagen deposition seemed restricted to the presumed top layers of the TIs, possibly as predefined by the in vitro differentiation step. At 8 weeks, type-II collagen deposition was either maintained (mineralized cartilage) or greatly decreased and replaced by type-I collagen (ossicles), suggesting cartilage remodeling and endochondral ossification (Fig. 3). Human osteocalcin-positive cells were found in both ossicles and mineralized cartilage, suggesting that implanted hPDCs partially contributed to de novo tissue formation. This variation could potentially be explained by a combination of factors, such as the variation between animals and a certain heterogeneity within the constructs after in vitro culture.

### Osteochondral regenerative potential of TIs demonstrated in critical size osteochondral defects

**Fig. 4:**
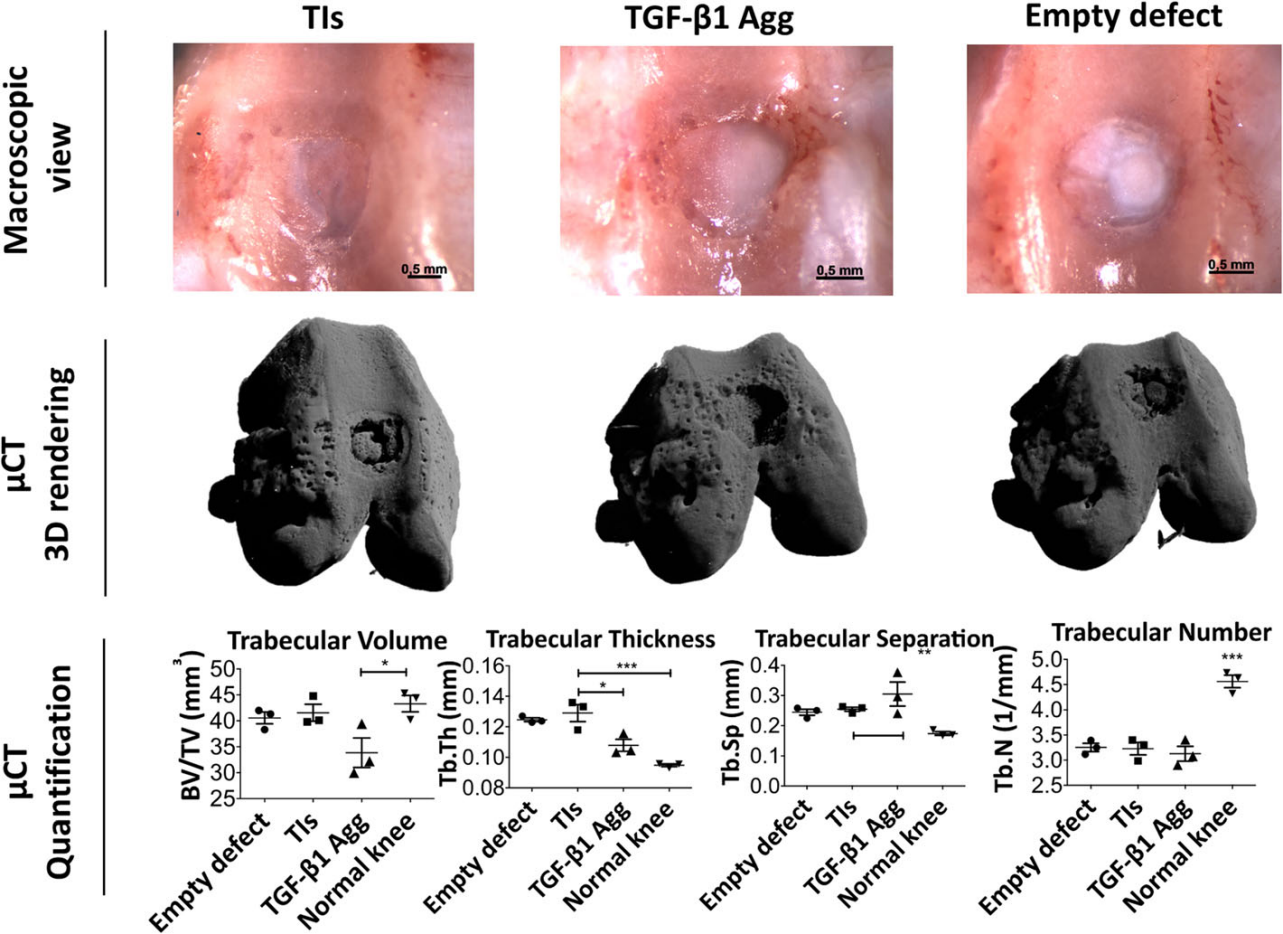
Macroscopic view, μCT 3D rendering, and bone quantification and characterization of treated and nontreated osteochondral defects at 8 weeks postinjury. Critical-size osteochondral defects (1 mm depth × 1.4 mm diameter) were treated with tissue intermediates (TIs) or transforming growth factor (TGF)-β1 aggregates (Agg). Empty osteochondral defects were used as negative controls. Macroscopic view of the osteochondral defects showed integration of the implanted constructs at the defect site and similar morphology of those receiving no treatment (empty defects). μCT 3D rendering showed subchondral bone regeneration at the osteochondral defect site. Quantification and characterization of new subchondral bone formation at the defect site was performed by analyzing a region of interest 1.5 mm diameter × 1 mm depth. *n* = 3 animals per condition. One-way ANOVA followed by Tukey’s multiple comparison test was used to analyze the results. *P < 0.05, **P < 0.01, ***P < 0.001

**Fig. 5:**
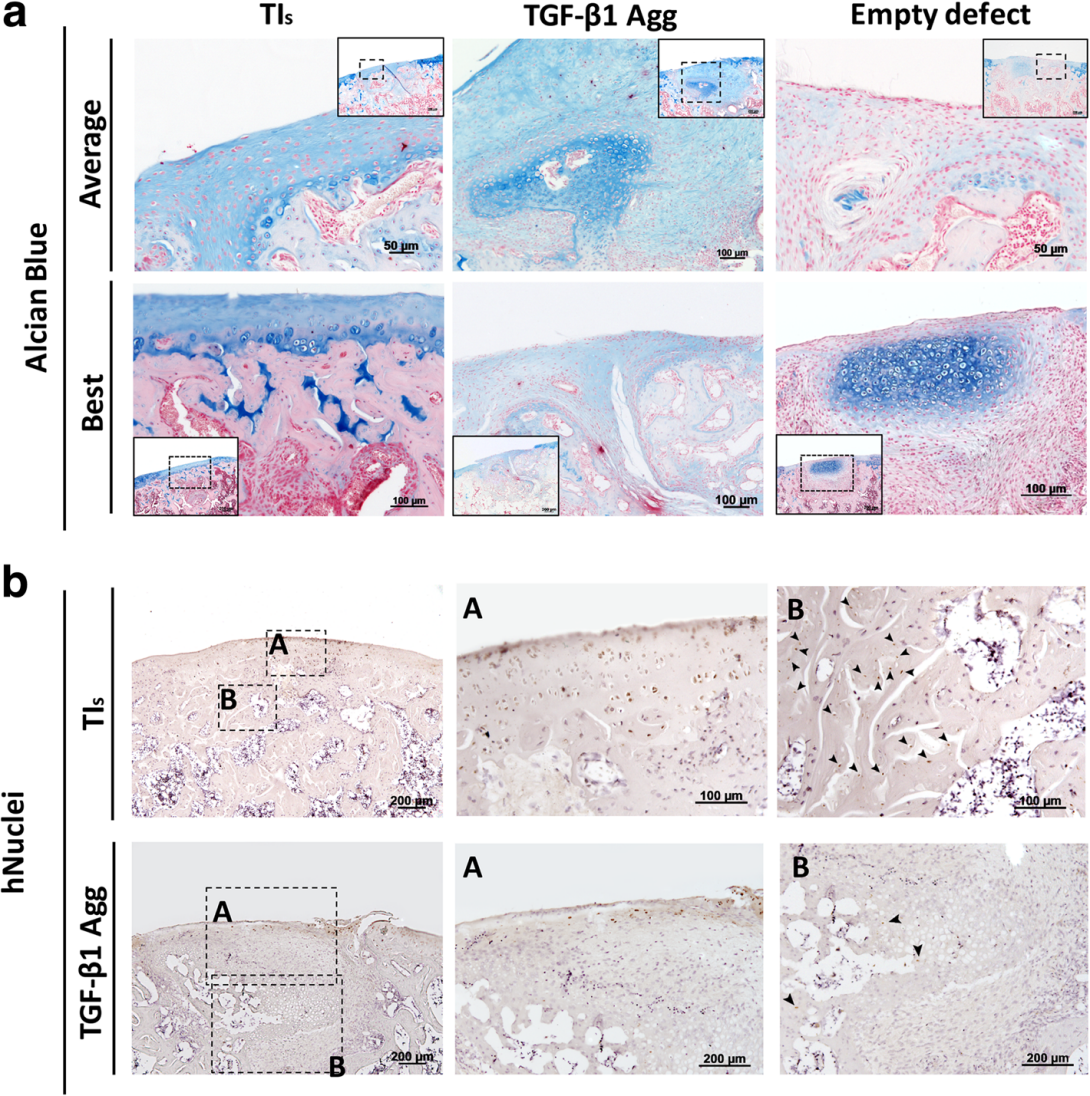
Histological analysis of osteochondral defects and human cell identification at 8 weeks postinjury. **a** Alcian blue staining showed articular-like tissue expressing GAGs in osteochondral defects treated with tissue intermediates (TIs), limited bone forming capacity at the subchondral level, and the presence of nonfunctional fibrotic tissue in osteochondral defects treated with transforming growth factor (TGF)-β1 aggregates (Agg), and limited capacity of empty defects to repair articular cartilage. The average and best histological sections are shown. **b** Immunohistochemistry for human (h)nuclei in defects treated with TIs and TGF-β1 aggregates showed the contribution of human cells (arrow heads) for both articular and subchondral bone regeneration

**Fig. 6:**
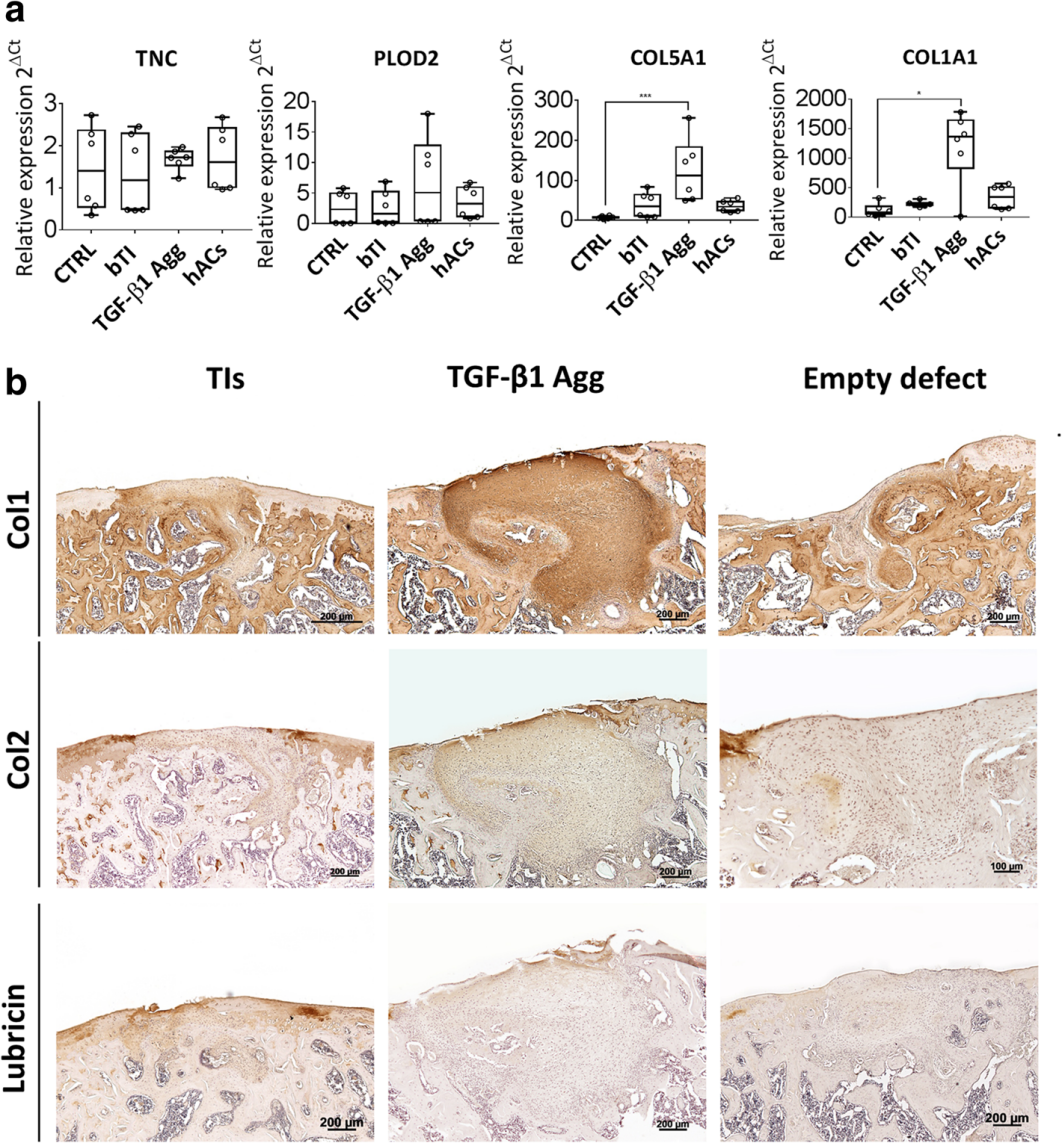
Characterization of tissue intermediates (TIs) or transforming growth factor (TGF)-β1 aggregates (Agg) at the gene expression level before implantation and histological analysis of type-I collagen (Col1), type-II collagen (Col2), and lubricin deposition at 8 weeks postinjury. **a** Gene expression analysis of fibrosis-related genes between conditions. **b** Immunohistochemistry showed homogeneous deposition of type-I collagen in osteochondral defects treated with TGF-β1 aggregates, as compared to empty defects and defects treated with TIs. Type-II collagen deposition is present in the pericellular space of cells in treated defects, but absent at the articular surface of empty defects. Immunohistochemistry for lubricin showed positive staining in treated osteochondral defects, but negative staining in empty defects. Histology performed in samples representing the average response. For gene expression, the results are representative of two independent experiments, each experiment was performed in triplicate; error bars are max/min. *P < 0.05, **P < 0.01, ***P < 0.001. CTRL control, hACs human articular chondrocytes, PLOD2 procollagen-lysine, 2-oxoglutarate 5-dioxygenase 2, TNC tenascin C

A translational model of osteochondral defect repair was used to test the functional properties of these TIs. Critical-size osteochondral defects (1.4 mm diameter × 1 mm depth) were created in the knees of nude rats and treated with TIs and TGF-β1 aggregates (cultured in BCM supplemented with 10 ng/ml TGF-β1 for 4 weeks). Empty defects served as the negative control. For subchondral bone characterization, a region of interest (ROI) of 1.5 mm diameter × 1 mm depth was drawn at the site of the defect and compared to a similar area in contralateral noninjured knees. At 8 weeks postinjury, macroscopic observation showed integration of the implanted constructs at the defect site, which morphologically resembled the empty defects (Fig. 4). μCT three-dimensional rendering showed the location of the defect and suggested a different range of subchondral bone regeneration between conditions (Fig. 4). Morphological analysis of the subchondral bone within the regenerating area showed that defects treated with TIs and empty defects displayed similar subchondral bone volume (BV/TV) and bone morphology (*P* > 0.05) (Fig. 4). Conversely, defects treated with TGF-β1 aggregates revealed limited subchondral bone forming capacity, as the BV/TV was significantly lower when compared to normal knees (*P* < 0.05). The increased BV/TV in defects treated with TIs and empty defects seemed to occur due to the appearance of thicker new trabecular bone, as showed by a significant increase of the trabecular thickness (Tb.Th) in these two conditions (*P* < 0.05). Analysis of trabecular separation (Tb.Sp) indicated that the new subchondral bone in defects treated with TIs and empty defects was similar to that of normal knees (*P* > 0.05), while defects treated with TGF-β1 aggregates showed significantly higher Tb.Sp (*P* < 0.01). The trabecular number was similar to those in all the experimental conditions, but significantly lower than that of normal knees (*P* < 0.001) (Fig. 4). Histological characterization showed heterogeneous osteochondral regeneration across the defects. Defects treated with TIs showed subchondral bone regeneration and newly formed cartilage at the joint surface at 8 weeks postinjury (Fig. 5a). In the average response, the newly formed cartilage tissue resembled immature cartilage as shown by alcian blue staining and polygonal chondrocyte-like cells. However, the best histological results suggested that TIs can contribute to fully regenerate the osteochondral unit. Conversely, defects treated with TGF-β1 aggregates displayed an accumulation of nonmineralized fibrocartilage at the putative location of the subchondral bone and articular cartilage (Fig. 6b), which may explain the delay/suppression of the subchondral bone regeneration observed in empty defects. The histological result with the highest level of repair for this condition showed sporadic subchondral bone regeneration, but the articular surface is fibrocartilage. To investigate the contribution of hPDCs to the newly formed tissues, human cells were tracked using an anti-human nuclei antibody. The results confirmed that hPDCs contributed to both articular cartilage and bone formation in defects treated with TIs, and to a lesser extent in the TGF-β1 condition (Fig. 5b).

### TGF-β1 induces in vitro fibrocartilage formation and suppresses bone forming capacity of hPDCs in osteochondral defects

To elucidate the mechanisms by which TIs facilitate bone and cartilage regeneration as compared with TGF-β1 aggregates, the expression of fibrosis-related genes between these two conditions was analyzed prior to implantation. Four fibrosis-related gene markers were analyzed by qRT-PCR, including Col1A1 [29], procollagen-lysine, 2-oxoglutarate 5-dioxygenase 2b (Plod2b) [30], Tenascin C (TNC) [31], and Col5A1 [32]. Additionally, SOX9 and RUNX2, as well as COL2A1 and COL10A1, were analyzed to assess chondrocyte differentiation and maturation between conditions (Additional file 3: Figure S2). Gene expression analysis indicated that PLOD2, COL5A1, and COL1A1 had a trend for increased expression in TGF-β1 aggregates when compared with TIs, whereas COL1A1 and COL5A1were significantly upregulated in TGF-β1 aggregates (*P* < 0.01) (Fig. 6a). TNC gene expression did not change between conditions. Maintenance of a fibrotic phenotype after orthotopic implantation was confirmed by immunohistochemistry for type-I collagen in defects treated with TGF-β1 aggregates. In contrast, defects treated with TIs and empty defects displayed limited type-I collagen deposition (Fig. 6b). To further study the functionality of the newly formed tissues between conditions, immunohistochemistry for type-II collagen and lubricin was performed. Both proteins were found in treated defects; however, TGF-β1 conditions displayed a fibroblast-like shape across their volume. In both cases, type-II collagen deposition was found at a lower extent when compared with native surrounding cartilage. In opposition, empty defects were void of type-II collagen deposition and lubricin expression.

## Discussion

The repair of osteochondral joint surface defects through MSC-based tissue engineering remains challenging. Previous studies have convincingly shown the positive effect of TGF-β (TGF-β1 and TGF-β3 specifically) on chondrocyte differentiation of multiple MSC types, including hPDCs [25, 33]. However, a single application of TGF-β proved insufficient to generate cartilage grafts with similar mechanical and biochemical properties to native articular cartilage [34, 35], and rather generated fibrocartilage [36]. In addition, only a few studies have attempted to repair osteochondral defects using three-dimensional MSC-based grafts in a scaffold-free experimental setting [37, 38].

In this work, it was hypothesized that TIs generated from hPDCs cultured in a novel developmentally inspired combination of GFs could respond to the stimuli of the surrounding microenvironment to generate synchronized tissues resembling the stable osteochondral unit of joints. When developmentally inspired differentiation conditions were applied, hPDCs generated bilayered constructs displaying osteochondral potential. At the molecular level, these constructs expressed genes associated with stable articular chondrocytes, such as SOX9, COL2A1, and NOGGIN, along with the hypertrophic genes MEF2C and COL10A1. Higher expression of NOGGIN in TIs as compared to hACs suggests the presence of endogenous mechanisms to control BMP signaling and avoid the potential hypertrophic differentiation effect of BMP signaling in TIs. Indeed, a significant lower expression of GREMLIN1 in TIs as compared with hACs suggests that NOGGIN is potentially the main regulator of the BMP pathway in this system. Surprisingly, control aggregates displayed an increased expression of PRG4, which raises the question of whether expanded hPDCs encompass a subpopulation of PRG4^+^ cells that can be maintained under specific culture conditions. Since PRG4 has recently been suggested as a potential marker of joint interzone progenitor cells [39], this observation might be of particular interest in the field of cartilage tissue engineering.

At the histological level, a time-dependent increase in IHH expression, cartilage ECM deposition (GAGs and type-II collagen), and establishment of different biological gradients from the top to the bottom cell layers of the TIs were found. This hierarchical organization of hPDC-derived chondrocytes, ranging from a dual type-II/type-I collagen-expressing zone to a type-I collagen-expressing zone, suggested an in vitro recapitulation of some aspects of the osteochondral differentiation. Based on previous studies, we hypothesized that different gradients of oxygen, nutrients, and GFs through the constructs and/or the contact with the plastic surface of the culture vessel might contribute to this self-patterning event [40–43]. Whilst it is unclear which of the constituents of the bi-layered construct are important for osteochondral repair, we believe the advantages are clear from the impressive tissue regeneration observed compared with other treatments.

It is noteworthy that ectopic implantation of TIs in nude mice showed either the generation of ossicles or “stable” mineralized cartilage. Considering that MSC-derived chondrocytes are permissive to cartilage remodeling and bone formation via endochondral ossification [44, 45], our findings may represent a significant advance towards the generation of articular cartilage tissue-engineered constructs. Also, both donor and host cells contributed to in vivo tissue formation, as showed by anti-human osteocalcin. Osteocalcin-positive cells are likely to contribute to the mineralization events within the cartilage matrix, as this protein acts as a nucleation site for hydroxyapatite deposition [46]. Osteocalcin expression can potentially be explained by the in vitro activation of the BMP pathway, which is known to contribute to cartilage mineralization [47]. Interestingly, the mineralized cartilage displayed type-II collagen deposition and seemed to be protected from vascular invasion and matrix remodeling, which suggested that the TIs were stabilized at a prehypertrophic chondrogenic state. Moreover, ectopic maintenance of structural type-II to type-I collagen gradients suggested an intrinsic capacity of the TIs to commit to an osteochondral differentiation process similar to that of the osteochondral unit. To test this hypothesis, a critical-size osteochondral defect model was used. To investigate the contribution of the orthotopic microenvironment and to separate that from the contribution of the chondrogenic medium used, cell aggregates cultured in basal chondrogenic medium supplement with 10 ng/ml TGF-β1 were also tested. TGF-β1 was chosen because of its known potential to induce chondrogenic differentiation of hPDCs [25]. Following 8 weeks of implantation in osteochondral defects, μCT analysis showed significant differences at the level of subchondral bone regeneration between conditions, especially in osteochondral defects treated with TGF-β1 aggregates where limited subchondral bone regeneration was observed. This correlated with an accumulation of fibrocartilage tissue in the subchondral bone area. Gene expression analysis prior to implantation indicated that chondrocyte differentiation through TGF-β1 triggers not only a significant increase in COL2A1 and COL10A1, but also a fibrotic tissue response involving Plod2, COL5A1, and COL1A1 [29–32]. These data corroborate previous studies that correlated the overexpression of TGF-β signaling with cartilage degradation in murine OA models [48, 49]. Nevertheless, TGF-β1 aggregates were able to express lubricin at the joint surface 8 weeks postinjury, possibly as a direct effect of TGF-β1 stimulation in superficial articular chondrocytes [50]. Defects treated with TIs displayed subchondral bone regeneration to levels similar to normal knees and empty defects, with human cells contributing to this process. At the joint surface, these defects displayed increased GAG, type-II collagen, and lubricin deposition, notably with human cells also contributing to these processes. These data indicate that periosteal cell expansion and differentiation under optimized conditions can generate an enhanced outcome when compared to autologous osteoperiosteal transplantation into osteochondral defects. Indeed, while osteoperiosteal transplantation improves osseointegration, concurrent deterioration of the overlying cartilage repair tissue is observed [51].

Despite the encouraging results provided herein, long-term follow-up is necessary to characterize the stability of the new cartilage-subchondral bone unit which was formed. Despite this, it is hypothesized that the regeneration of the subchondral bone is likely to contribute to the stability of the newly formed cartilage layer, as early subchondral bone loss is thought to contribute to OA initiation.

## Conclusions

In summary, we report the potency of a novel GF cocktail for osteochondral differentiation of expanded hPDCs. This GF cocktail allows either progression or stabilization of the cartilage phenotype in a microenvironment-dependent manner, supporting the hypothesis that a scaffold-free tissue-engineered construct from hPDCs can be engineered in vitro for treatment of osteochondral defects. This apparent plasticity allows the osteochondral-like implants described herein to have applications in treating focal chondral lesions and full thickness osteochondral defects, thus potentially reducing the incidence of OA from the current estimate of 15% of all adults aged over 60 years.

## Additional files

Additional file 1: **Table S1.** Details of the antibodies and immunohistochemistry protocols employed. (DOCX 17 kb)

Additional file 2: **Figure S1.** Early mineralization patterns of TIs at 2 weeks after ectopic implantation in nude mice. Left: alcian blue staining showing the presence of mineralized cartilage in the top layers of the construct (encircled by dotted black lines) and vascular ingrowth from the bottom layers (black arrows). Right: μCT cross-section confirming the presence of mineralized tissues (encircled by dotted black lines) and nonmineralized GAG-positive tissues, between white and black lines (Visualization of nonmineralized cartilage was possible following incubation with Hexabrix, a cartilage contrast agent). (TIFF 14998 kb)

Additional file 3: **Figure S2.** Gene expression analysis of chondrogenesis in CTRL, TIs, TGF-β1 aggregates, and hACs after 28 days in culture. Results are representative of two independent experiments, each experiment was performed in triplicate. error bars are max/min; *P < 0.05, **P < 0.01, ***P < 0.001. (TIFF 240 kb)

## Abbreviations

ACI: Autologous chondrocyte implantation
BCM: Basal chondrogenic medium
BMP: Bone morphogenetic protein
BSA: Bovine serum albumin
CTRL: Control
DMEM: Dulbecco’s modified Eagle’s medium
ECM: Extracellular matrix
FBS: Fetal bovine serum
FGF: Fibroblast growth factor
GAG: Glycosaminoglycan
GDF: Growth differentiation factor
GF: Growth factor
H&E: Hematoxylin and eosin
H_2_O_2_: Hydrogen peroxide
hACs: Human articular chondrocytes
hPDC: Human periosteum-derived progenitor cell
HRP: Horseradish peroxidase
IHH: Indian Hedgehog
MSC: Mesenchymal stem cell
OA: Osteoarthritis
PBS: Phosphate-buffered saline
PFA: Paraformaldehyde
qRT-PCR: Quantitative reverse transcription polymerase chain reaction
ROI: Region of interest
TBST: Tris-buffered saline and 0.1% Tween 20
TGF: Transforming growth factor
TI: Tissue intermediate
μCT: Microcomputed tomography
