# Supplementary material for: Advancing osteochondral tissue engineering: bone morphogenetic protein, transforming growth factor, and fibroblast growth factor signaling drive ordered differentiation of periosteal cells resulting in stable cartilage and bone formation in vivo
Source: Stem Cell Res Ther. 2018 Feb 21;9:42. doi: 10.1186/s13287-018-0787-3 (PMC5822604; doi:10.1186/s13287-018-0787-3)
Supplement: Supplementary file 1 — Table S1. Details of the antibodies and immunohistochemistry protocols employed. (DOCX 17 kb) [file 13287_2018_787_MOESM1_ESM.docx]

## Supplementary information

**Additional file 1: Table S1** – Details of the antibodies and immunohistochemistry

protocols employed

| *Antibody* | *Manufacturer* | *Antigen Retrieval* | *Block* | *2^ndary^ antibody* |
| --- | --- | --- | --- | --- |
| COL2 | abcam | Pepsin, RT, 15 min | 5% BSA, PBS + 0.1%Tween20  +  0.01%TritonX | HRP-conjugated anti rabbit,  Jackon |
| COL1 | ThermoFisher Scientific | Pepsin, RT, 15 min | 5% BSA, PBS + 0.1%Tween20  +  0.01%TritonX | HRP-conjugated anti-rabbit,  Jackson |
| PRG4 | abcam | Hyaluronidase  37 ˚C, 30 min | 5% BSA, TBS  +  0.1% Tween20  +  0.01% TritonX | HRP-conjugated anti-rabbit, Jackson |
| Osteocalcin | KULeuven | Citrate, 98 ˚C, 15 minutes | 5% BSA, TBS  +  0.1% Tween20  +  0.01% TritonX | HRP-conjugated anti-guinea pig, Jackson |
| Human Nuclei | Millipore | 0.5% Triton PBS, 15 min  +  Proteinase K, 20 minutes | 5% BSA, TBS  +  0.1% Tween20  +  0.01% TritonX | HRP-conjugated anti-mouse, Jackson |

**RT:** Room temperature; **BSA**: Bovine Serum Albumine; **PBS**: Phosphate buffered saline. **Citrate buffer:** 10mM citrate, buffer adjusted to pH 6; **Hyaluronidase:** bovine hyaluronidase 450 IU/mg, 4 mg/ml adjust to pH 5 with 0.1M HCl; **Pepsin:** 0.1% pepsin in 10nM HCL pH2; **Proteinase K:** Proteinase K solution 0.6 units/mL in TE buffer (50mM Tris Base, 1 mM EDTA, 0,5% Triton X-100), pH=8.
