# Supplementary figures and images for: Advancing osteochondral tissue engineering: bone morphogenetic protein, transforming growth factor, and fibroblast growth factor signaling drive ordered differentiation of periosteal cells resulting in stable cartilage and bone formation in vivo
Source: Stem Cell Res Ther. 2018 Feb 21;9:42. doi: 10.1186/s13287-018-0787-3 (PMC5822604; doi:10.1186/s13287-018-0787-3)

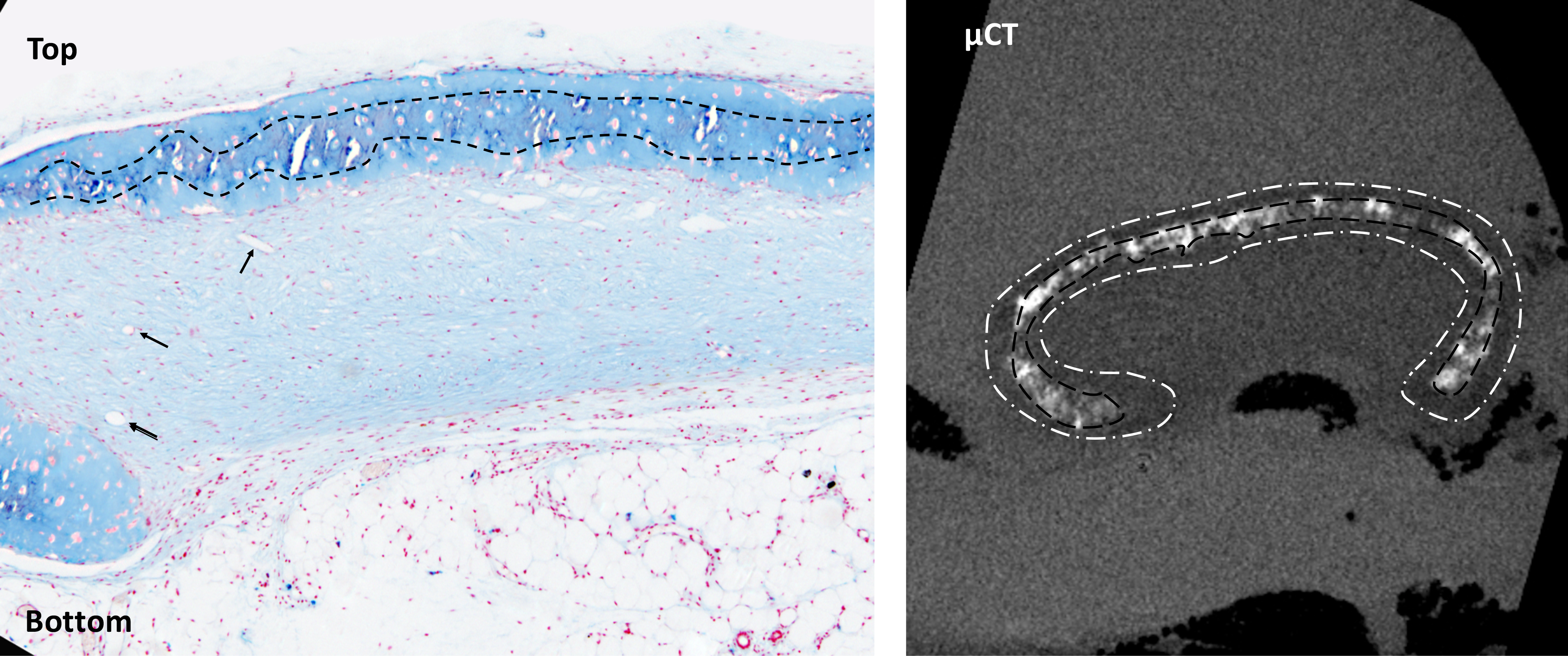

Supplement: Supplementary file 2 — Figure S1. Early mineralization patterns of TIs at 2 weeks after ectopic implantation in nude mice. Left: alcian blue staining showing the presence of mineralized cartilage in the top layers of the construct (encircled by dotted black lines) and vascular ingrowth from the bottom layers (black arrows). Right: μCT cross-section confirming the presence of mineralized tissues (encircled by dotted black lines) and nonmineralized GAG-positive tissues, between white and black lines (Visualization of nonmineralized cartilage was possible following incubation with Hexabrix, a cartilage contrast agent). (TIFF 14998 kb) [file 13287_2018_787_MOESM2_ESM.tif]

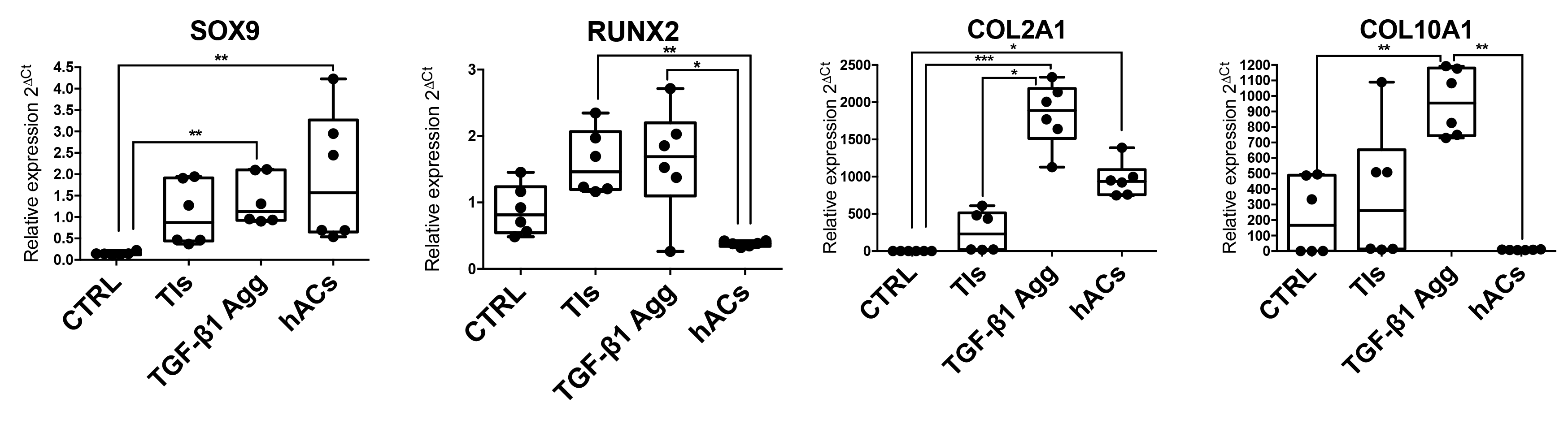

Supplement: Supplementary file 3 — Figure S2. Gene expression analysis of chondrogenesis in CTRL, TIs, TGF-β1 aggregates, and hACs after 28 days in culture. Results are representative of two independent experiments, each experiment was performed in triplicate. error bars are max/min; *P < 0.05, **P < 0.01, ***P < 0.001. (TIFF 240 kb) [file 13287_2018_787_MOESM3_ESM.tif]
